# Biombalance^TM^: A Specific Oligomeric Procyanidin-Rich Grape Seed Extract as Multifunctional Ingredient Integrating Antibacterial, Antioxidant, and Anti-Inflammatory Activities with Beneficial Gut–Brain Axis Modulation

**DOI:** 10.3390/antiox14121484

**Published:** 2025-12-10

**Authors:** Mohamed Mokrani, Amandine Brochot, Maria C. Urdaci

**Affiliations:** 1 University of Bordeaux, CNRS, Bordeaux INP, CBMN, UMR 5248, F-33600 Pessac, France; 2 Bordeaux Sciences Agro, F-33175 Gradignan, France; 3 Groupe Berkem, 20 Rue Jean Duvert, F-33290 Blanquefort, France; amandine.brochot@berkem.com

**Keywords:** oligomeric procyanidin (OPCs), grape seed extract, antimicrobial, antioxidant, anti-inflammatory, microbiota, gut–brain axis, gut homeostasis, glutamate, NPY, GLP-1, GLP-1r

## Abstract

Polyphenols, as natural compounds abundant in plant-derived foods, have been recognised for their human health benefits. This study evaluates the multifunctional properties of Biombalance^TM^ (BB), a grape seed extract rich in oligomeric procyanidins, in various in vitro and in vivo models. BB was studied to assess (i) its antimicrobial effects in different bacterial species; (ii) its protective effects against oxidative and inflammatory stress in Caco-2 cells; and (iii) its effects in mice, which were fed a standard diet with or without BB at two different doses (BB1X and BB2X) to understand the impacts of BB on microbiota and gut homeostasis. BB selectively inhibited several bacterial species, including *Staphylococcus aureus*, *Helicobacter pylori*, and *Blautia coccoides*. In addition, BB protected Caco-2 cells against hydrogen peroxide (H_2_O_2_)-induced oxidative damage and lipopolysaccharide (LPS)-induced oxidative and inflammatory stress. In vivo, BB supplementation upregulated the expression of antioxidant and homeostasis genes in the colon, ileum, and liver, accompanied by dose-dependent changes in the gut microbiota composition. Functional predictions indicated favourable modulation of microbial metabolic pathways, including those involved in antioxidant capacity and glutamate degradation. Furthermore, BB positively influenced key gut–brain axis mediators, including GLP-1, the GLP-1 receptor, and NPY. These findings highlight the potential of Biombalance^TM^ to support health and gut–brain communication and to protect against oxidative and inflammatory stress in the gut.

## 1. Introduction

Plant secondary metabolites, such as alkaloids, terpenoids, and phenolic compounds (polyphenols) help plants to survive in a competitive environment [1]. Polyphenols are defined by the presence of one or more hydroxyl (-OH) groups connected to an aromatic benzene ring [2]. Flavonoids, an essential class of polyphenols, comprise more than 6000 different compounds divided into 12 groups, ranging from simple phenolic acids to complex tannins [3]. This family includes, among others, flavanones, flavonols, procyanidins, and anthocyanins. Proacyanidins—also known as oligomeric procyanidins (OPCs) are a group of condensed tannins formed by flavan-3-ol subunits, such as catechin and epicatechin, linked by interflavan bonds of A-type or B-type [4].

Thanks to this chemical structure, OPCs have a high antioxidant potential, allowing them to decrease oxidative stress by neutralising free radicals, and also exhibit potent anti-inflammatory properties by modulating several inflammatory pathways. Oxidative stress and inflammation play significant roles in a wide range of chronic diseases, including cardiovascular disorders, metabolic diseases, cancers, and neurodegenerative conditions [3,5,6].

Another notable feature of OPCs is their antimicrobial properties, which play a crucial role in controlling the invasion of plant pathogenic microorganisms [7]. These antibacterial effects observed in plants have also been established in other kingdoms. For example, OPCs can inhibit the growth of human and animal pathogens, including *Staphylococcus aureus*, *Prevotella* spp., *Clostridium* spp., *Porphyromonas gingivalis*, and *Fusobacterium nucleatum* [8,9]. OPCs differ significantly in their chemical structures from other classes of polyphenols, presenting complex structure–activity relationships. The most studied proanthocyanidins are those from berries, and they have been shown to inhibit the growth of several pathogenic bacteria, such *Staphylococcus aureus*, the cariogenic *Streptococcus mutans*, and the uropathogenic *Escherichia coli* [10]. Regarding the activities of grape extracts, studies have shown that their anti-*Helicobacter pylori* activity depends on the part of the plant they are extracted from, the grape variety, and the synergistic mode of action of the extracted compounds [11]. Moreover, the antimicrobial properties of OPCs could have benefits for inhibiting small intestinal bacterial overgrowth (SIBO), which leads to bacterial overgrowth and dysbiosis in the small intestine [12].

On the other hand, some studies have demonstrated that polyphenols have prebiotic effects [13] and that grape seed OPCs can stimulate the growth of beneficial bacteria, such as *Lactobacillus* and Bifidobacteria [14,15]. Overall, the beneficial effects of polyphenols and polyphenol–gut interactions known at present are not only restricted to GIT disorders but have also been associated with extra-digestive effects. Emerging research has highlighted OPCs as promising candidates for modulating neuroinflammatory and age-associated processes, and they have been tested for their ability to mitigate neurodegenerative diseases [16]. As most OPCs are poorly absorbed, and their liver-derived metabolites are unable to cross the blood–brain barrier, it has been suggested recently that the interactions of polyphenols with the enteric nervous system, vagus nerve, and the gut microbiota contribute to their extra-digestive bioactivities, notably in the so-called gut–brain axis paradigm [17,18,19]. OPCs are the primary active polyphenolic compounds in BB. Few investigations have explored the differences in bioactivity between red and white grape seed extracts and the effects of different doses on health outcomes [20]. In a previous study, we presented an integrative analysis of BB’s multi-target effects on colitis pathogenesis—involving microbiome shifts, immune regulation, and gut barrier restoration—thus providing a systems-level understanding of its potential in a colitis mouse model [21].

BB presents a high flavanol content and, similarly to cocoa, many of its health benefits are likely due to this high flavanol content (Supplementary Table S1) [22]. BB is composed of flavanol monomers and oligomers (procyanidins) [21]. The principal monomers identified were catechins (12.5%) and epicatechins (8.5%); dimer procyanidins (18.9%) were represented by four types (B1, B2, B3, and C1), where B-type procyanidin dimers accounted for approximately 80% of the total procyanidins; and trimers (6.1%), tetramers (3%), and DP5 to TP7 forms (1.6%) were also present [21].

In the present study, we provide new evidence for the bioactivity of the grape seed extract BB through various molecular pathways, particularly highlighting its antimicrobial properties, roles in oxidative stress and inflammatory responses, and modulation of microbiota in both in vitro and in vivo studies.

## 2. Materials and Methods

### 2.1. Bacterial Strains and Growth Conditions

The bacterial strains *Listeria innocua* CIP 106065, *Listeria monocytogenes* CIP 82110, *Erwinia carotovora* CECT 225, *Pseudomonas aeruginosa* CIP 76110, and *Kocuria rhizophila* ATCC 10240 were cultivated aerobically at 30 °C in TS medium (BD, Le Pont-de-Claix, France) for 24 h. *Staphylococcus aureus* CIP 20256, *Streptococcus mutans* ATCC 35668, *Enterococcus faecalis* CIP 76117, and *Escherichia coli* CIP 7624 were grown under identical aerobic conditions at 37 °C. *Lactiplantibacillus plantarum* 299v and *Pediococcus acidilactici* DSM 20284 were grown in IST medium (Difco, Le Pont-de-Claix, France) at 30 °C.

*Blautia coccoides* DSM 935, *Porphyromonas gingivalis* ATCC 33277, *Fusobacterium nucleatum* ATCC 25586, and *Cutibacterium acnes* DSM 1897 were cultured anaerobically at 37 °C in modified MCMM medium, and *Akkermansia muciniphila* DSM 22959 in PYG medium using a Bactron anaerobic chamber (Cornelius, OR, USA) for 48 h. *Helicobacter pylori* strains P12 and 7.13 were maintained on Columbia agar supplemented with 10% horse blood under microaerophilic conditions (5–10% O_2_, 5–10% CO_2_) at 37 °C for 48 h [23]. All strains were propagated according to species-specific requirements to ensure optimal growth and metabolic activity.

### 2.2. Bacterial Minimum Inhibitory Concentration

#### 2.2.1. Bacterial Minimum Inhibitory Concentration in Aerobic Conditions

BB was assessed for inhibitory effects against the strains *Listeria innocua* CIP 106065, *Listeria monocytogenes* CIP 82110, *Kocuria rhizophila* ATCC 10240, *Erwinia carotovora* CECT 225, *Pseudomonas aeruginosa* CIP 76110, *Staphylococcus aureus* CIP 20256, *Streptococcus mutans* ATCC 35668, and *Escherichia coli* CIP 7624 using a 96-well microdilution (Corning, Kennebunk, ME, USA) (n = 3 for every condition) method, in alignment with EFSA [24] and CLSI standards [25]. Serial dilutions of BB (32–0.125 mg/mL) in adequate, standardised medium were added to 5 × 10^5^ CFU/mL, equivalent to 0.5 McFarland dilution. After aerobic incubation (35 °C, 18–24 h), the OD 600 nm was measured in a Multiskan FC photometer (Thermo, Vantaa, Finland) to define the MICs. Gram staining of cultures was performed as a control. All tests were performed in triplicate to ensure reproducibility.

#### 2.2.2. Bacterial Minimum Inhibitory Concentration in Anaerobic Conditions

BB was assessed for inhibitory effects against *Enterococcus faecalis* CIP 76117, *Blautia coccoides* DSM 935, *Porphyromonas gingivalis* ATCC 33277, *Fusobacterium nucleatum* ATCC 25586, and *Cutibacterium acnes* DSM 1897 using the 96-well microdilution (Corning, OR, USA) method, as mentioned before, except for anaerobic incubation (35 °C, 48–72 h) in an anaerobic incubator (Bactron, OR, USA). All tests were performed in triplicate to ensure reproducibility.

#### 2.2.3. Bacterial Minimum Inhibitory Concentration Microaerophilic Conditions

*Helicobacter pylori* was cultured from clinical strains on Columbia blood agar supplemented with 5–10% horse blood and incubated under microaerophilic conditions (5% O_2_, 10% CO_2_, 85% N_2_) at 37 °C for 2–3 days. Colonies were harvested and suspended in sterile distilled water or broth to a turbidity equivalent to a 3.0 McFarland standard (approximately 5 × 10^8^ CFU/mL). Using a sterile swab, the suspension was evenly spread over the surface of Mueller–Hinton agar with 5–10% horse blood [23]. After the plate dried, a gradient of BB (32–0.125 mg/mL) was impregnated and applied to the agar. Plates (n = 3) were incubated at 35–37 °C in a microaerophilic atmosphere for 3–5 days. The minimum inhibitory concentration (MIC) was determined at the point where the inhibition ellipse intersected the test strip, with the MIC defined as the lowest concentration of BB that prevented visible growth. All tests were performed in triplicate to ensure reproducibility.

### 2.3. Caco-2 Cells Culture

Caco-2 human colon cancer cells (ATCC, HTB-37), kindly provided by Prof. Christine Varon, were used. Cells were grown in DMEM (Sigma, Taufkirchen, Germany) supplemented with 10% FBS (Sigma, Cajamar, Brazil) and 1% penicillin and streptomycin (Sigma, Taufkirchen, Germany), and were maintained in an atmosphere of 5% CO_2_ at 37 °C. Cells were cultured in a culture flask with medium changes every two days until they reached 90% confluence. Then, they were digested with 0.25% trypsin (Sigma, Taufkirchen, Germany) at 37 °C for 1–2 min and seeded in a six-well cell culture plate (Corning Costar, NY, USA) to culture for further experiments. The Caco-2 cells were maintained in culture for 21 days, with medium changes every 2 days, until complete cellular differentiation was achieved.

### 2.4. Study of the Antioxidant Effect of BB in Caco-2 Cells

Based on preliminary MTT assay results, a dose–response analysis was performed in which Caco-2 cells were incubated with BB diluted entirely in sterile distilled water and then added to the medium at concentrations ranging from 1 to 100 μg/mL, in order to evaluate its cytotoxicity and select biologically active doses.

For these preliminary tests, 5 μg/mL (BB1X) and 10 μg/mL (BB2X) concentrations were selected for 24 h incubation in the preventive treatment groups. Cells in both positive and negative control groups were incubated with standard medium alone [26]. To test for antioxidant effects, various groups were created. In the negative control (Ctl), untreated cells were maintained in standard medium without the addition of H_2_O_2_. In the treatment control (TC), the standard culture medium was supplemented with 1 mM of H_2_O_2_ for 4 h. To observe a potential effect of the BB alone in the Caco-2 cells, the cells of the BB1X group were supplemented with 5 μg/mL of BB, those of the BB2X group with 10 μg/mL of BB, and then the cells were incubated for 24 h in standard medium without H_2_O_2_. The groups H_2_O_2_1X and H_2_O_2_2X were supplemented with 5 or 10 μg/mL of BB, respectively, for 24 h, and then maintained in standard medium supplemented with 1 mM of H_2_O_2_ for 4 h.

The percentage of cell viability was measured using the MTT 3-(4,5-dimethylthiazol-2-yl)-2,5-diphenyltetrazolium bromide assay, in order to evaluate the cytoprotective effect of BB against oxidative stress caused by the addition of H_2_O_2_ in the cultured Caco-2 cells [27]. The expression of genes related to oxidation (*CAT*, *HO1*, *SOD*, *iNOS*, and *NQO1*) was analysed via RT-qPCR.

### 2.5. Study of the Anti-Inflammatory Effect of BB in Caco-2 Cells

In the preventive treatment groups, Caco-2 cells were pre-incubated with BB grape seed extract diluted in sterile distilled water and then added to the medium at concentrations of 5 μg/mL and 10 μg/mL before the inflammatory challenge. This pre-treatment was performed with the aim of evaluating the preventive or protective effects of BB against inflammation induced by lipopolysaccharide (LPS). After the pre-incubation period, cells were exposed to 100 ng/mL LPS for 4 h to induce an inflammatory response [28].

To analyse the protective effect of BB against LPS treatment, we studied the expression of genes related to inflammation (*IL6*, *IL10*, *NFKB*) and tight junction proteins (*OCCLU* and *ZO1*).

### 2.6. Quantification of Gene Expression in Caco-2 by qRT-PCR

Total RNA extraction was performed with the RNeasy Mini Kit (QIAGEN, Hilden, Germany), followed by DNase I treatment (Turbo DNA-free^TM^, Thermo Fisher Scientific, Waltham, MA, USA) to eliminate genomic DNA contamination, by strictly adhering to the supplier’s protocols. Reverse transcription of 250 ng total RNA was performed using SuperScript^TM^ IV Reverse Transcriptase (Thermo Fisher Scientific, Vilnius, Lithuania). Quantitative PCR (qPCR) was conducted on a CFX96 Real-Time PCR System (Bio-Rad, Hercules, CA, USA) with iTaq^TM^ Universal SYBR^®^ Green Supermix (Bio-Rad). Each reaction was run in triplicate, and a standard curve was generated for each primer pair to determine the amplification efficiency. Gene expression levels were normalised to the housekeeping gene *β-actin* (*ACTB*), and relative expression was calculated using the 2^−ΔΔCT^   method. 

### 2.7. Animal Experiment Design

All procedures involving animals were conducted in compliance with French regulations (Rural and Maritime Fishing Code Articles R.214-87 to R.214-126) and approved by the Ethics Committee of Bordeaux University (APAFIS #48103-2024021616134560, October 2024).

Male Balb/c SFP-grade mice (6 weeks old) were sourced from Janvier Labs (Lorient, France) and housed under controlled conditions: 22 °C, 60% humidity, and a 12 h light/dark cycle. Mice received environmental enrichment (e.g., gnawing sticks, climbing structures) to support natural behaviours and had ad libitum access to water and a standard diet (SD, A04).

After a 7-day acclimatisation period, mice were randomly divided into three groups, with every group in a distinct cage (n = 8/group): control (Ctl), fed the standard A04 diet; BB1X, fed the A04 diet supplemented with 1 g/kg BB; and BB2X, fed the A04 diet supplemented with 2 g/kg BB. Feed and water consumption were verified daily throughout the experiment. The daily consumption of BB for the BB1X group was 2.3 ± 0.26 mg of BB per day. The daily consumption of BB for the BB2X group was 4.8 ± 0.18 mg of BB per day.

Daily health assessments were conducted to detect signs of distress or illness. Handling followed refined techniques to minimise stress, and analgesic protocols were implemented for any invasive procedures.

### 2.8. Tissue Sampling, RNA Extraction, and Real-Time PCR (RT-PCR)

Tissue samples from the ileum, colon, and liver were placed in RNAlater (QIAGEN, Hilden, Germany) and kept at 4 °C for 24 h to stabilise RNA. After this period, samples were transferred to −20 °C for storage until RNA extraction. Total RNA was extracted using the RNeasy Mini Kit (QIAGEN, Hilden, Germany) with an additional DNase treatment (Thermo Scientific, Vilnius, Lithuania). RNA concentrations were measured using a NanoDrop One spectrophotometer (Thermo Fisher Scientific, Wilmington, DE, USA), according to the manufacturer’s protocols. For cDNA synthesis, 250 ng of RNA was reverse-transcribed using random hexamers and the SuperScript IV system (Thermo Scientific, Vilnius, Lithuania). Quantitative PCR was performed on a CFX96 instrument (Bio-Rad, Hercules, CA, USA) employing iTaq^TM^ Universal SYBR Green Supermix (Bio-Rad, Hercules, CA, USA). Each reaction was run in triplicate, and a standard curve was generated for each primer pair to determine amplification efficiency. Gene expression levels were normalised to the housekeeping gene *HPRT*, and relative expression was calculated using the 2^−ΔΔCT^ method. The primer sequences and PCR cycling conditions are as detailed in our previous paper [21]. 

### 2.9. Stool Sampling, Faecal DNA Extraction, and 16S rDNA Sequencing

Faecal samples were collected in sterile tubes 24 h before the end of the experiment and stored at −80 °C. Approximately 100 mg of stool was homogenised in Tris-EDTA buffer (0.1 mM Tris, pH 8; 1 M EDTA; 1 mL buffer per 200 mg faeces), and lysozyme (300 mg/mL, Sigma, St. Louis, MO, USA) was added at a 1:100 ratio, followed by incubation at 37 °C for 1 h. DNA was then extracted from 300 μL of the mixture using the NucleoSpin^®^ Soil Kit (Macherey-Nagel, Düren, Germany) according to the manufacturer’s instructions, and quantified with a NanoDrop One spectrophotometer (Thermo Fisher Scientific, Wilmington, DE, USA) [29].

### 2.10. PCR and Microbiota Analysis

The V3–V4 region of the 16S rRNA gene was amplified using primers 338F and 806R, with PCR performed using the MP Taq DNA Polymerase kit (MP Biomedicals, Illkirch, France) under standard cycling conditions [30]. The PCR protocol involved an initial denaturation at 95 °C for 5 min, followed by 30 cycles comprising 30 s at 95 °C, 30 s at 52 °C for primer binding, and 45 s at 72 °C for strand elongation; the procedure concluded with a final elongation step at 72 °C for 2 min. Amplicons were sequenced on an Illumina MiSeq platform (Genotoul, Toulouse, France), and sequence quality was assessed using GALAXY FROGS 4.1. A total of 466,284 sequences were obtained after sequencing. Paired-end joined sequence reads were clustered into OTUs, which were further clustered using Swarm 4.1. Chimaeras, as well as OTUs representing less than 0.005% of total sequences, were removed [30]. Taxonomic assignment was performed using the SILVA 138 database, and data were normalised with DESeq2 1.38.3. Diversity indices, differential abundance analysis, and principal coordinate analysis (PCoA) were conducted using the SHAMAN software [31,32,33].

### 2.11. PICRUS (Phylogenetic Investigation of Communities by Reconstruction of Unobserved States) Analysis

PICRUSt2 (version 2.4) was applied to the 16S rRNA gene sequencing data generated via FROGS to investigate the metabolic capabilities of the microbiota. This computational tool predicts functional potential by inferring MetaCyc pathways and enzyme commission (EC) gene families from taxonomic profiles. The analysis prioritised third-tier pathway annotations (e.g., biosynthesis, degradation) to identify group-specific metabolic differences. Pathway abundances were derived by mapping EC numbers to the MetaCyc database—a comprehensive open-access metabolic pathway resource, which was chosen over KEGG due to its transparency and community-driven curation. This approach enabled the systematic identification of enzymatic reactions and metabolic networks linked to microbial community functions [34].

### 2.12. Statistical Analysis

Statistical analysis was performed using the GraphPad Prism software (version 10.01). Data were analysed using two-way analysis of variance (ANOVA), followed by Tukey’s multiple comparison tests, and the results are expressed as the mean ± standard deviation. A *p*-value less than 0.05 was considered significant.

Spearman’s rank correlation analysis was performed to assess the relationships between the relative abundances of *Blautia*, *Desulfovibrio*, and *Mucispirillum* and the expression levels of ileal genes. For continuous variables exhibiting significant correlations, the strength and direction of these associations were further examined.

## 3. Results

### 3.1. Antibacterial Effects of BB

The results demonstrate variable susceptibility of the diverse bacterial species tested to BB (Table 1), revealing three distinct MIC patterns: highly susceptible bacteria (*Kocuria rhizophila*, *Staphylococcus aureus*, and *Helicobacter pylori* P12 and 7.13 strains) with MIC values between 1 and 2 mg/mL; moderately susceptible species, including *Blautia coccoides* with an MIC of 8 mg/mL; and species presenting a lower susceptibility, with an MIC of 16 mg/mL (*Erwinia carotovora*, *Pseudomonas aeruginosa*, *Listeria innocua*, *Listeria monocytogenes*, *Streptococcus mutans*, *Enterococcus faecalis*, and *Porphyromonas gingivalis*). Moreover, some bacteria were not inhibited by high BB concentrations (MIC values higher than 32 mg/mL), including the probiotic bacteria *Lactiplantibacillus plantarum* 299v, *Pediococcus acidilactici*, and *Akkermansia muciniphila*, as well as the potential pathogens *Escherichia coli* and *Cutibacterium acnes* (formerly known as *Propionibacterium acnes*) [35,36].

### 3.2. Antioxidant Effect of BB In Vitro

**Figure 1:**
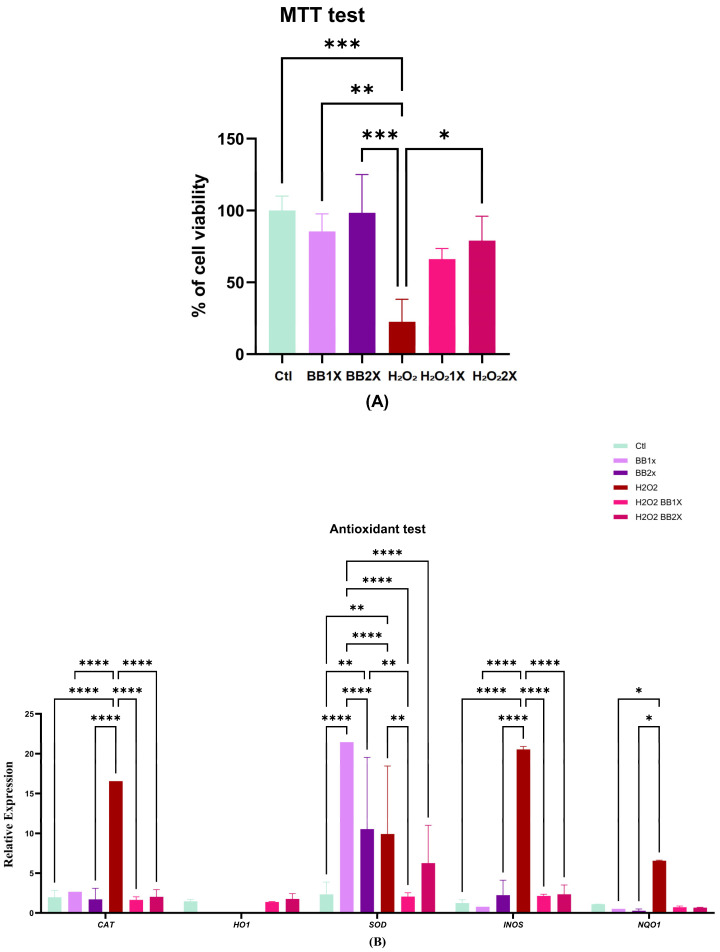
(**A**) MTT assay results showing cell viability in control (Ctl), BB1X, BB2X, H_2_O_2_ alone, and H_2_O_2_ with BB pre-treatment H_2_O_2_1X, H_2_O_2_2X. Data are mean ± SEM, n = 3. BB1X (5 µg/mL) and BB2X (10 µg/mL). * *p* < 0.05, ** *p* < 0.01, *** *p* < 0.001. (**B**) Relative gene expression of catalase (*CAT*), heme oxygenase-1 (*HO1*), superoxide dismutase (*SOD*), inducible nitric oxide synthase (*iNOS*), and NAD(P)H:quinone oxidoreductase 1 (*NQO1*) in Caco-2 cells: control (Ctl); cells treated with BB at 1X (BB1X) or 2X (BB2X) concentration; hydrogen peroxide (H_2_O_2_); and H_2_O_2_ with BB1X (H_2_O_2_ + BB1X) or H_2_O_2_ with BB2X (H_2_O_2_ + BB2X). Data are presented as mean ± SEM, n = 3. BB1X (5 µg/mL) and BB2X (10 µg/mL). Statistical significance is indicated as * *p* < 0.05, ** *p* < 0.01, and **** *p* < 0.0001.

The MTT assay was employed to evaluate the cytoprotective effect of BB at two concentrations (5 μg/mL and 10 μg/mL) against H_2_O_2_-induced oxidative damage in Caco-2 cells (Figure 1A). Cell viability in the control group (Ctl) was normalised to 100%, against which all other treatments were compared. Pre-treatment with BB at 5 μg/mL (1X) and 10 μg/mL (2X) for 24 h did not exhibit significant cytotoxicity compared to control cells, as indicated by viability values. Exposure to 1 mM H_2_O_2_ significantly reduced cell viability to 22.58 ± 15.3% (** *p* < 0.001 vs. control). Notably, pre-treatment with BB demonstrated significant dose-dependent protective effects against H_2_O_2_ challenge, with the H_2_O_2_1X and H_2_O_2_2X (* *p* < 0.05) groups exhibiting viability values of 66.12% ± 3.5% and 79.9% ± 6.5%, respectively. The gene expression graph (Figure 1B) demonstrates the modulatory effects of BB on oxidative stress and antioxidant gene expression in the hydrogen peroxide (H_2_O_2_) challenge model. In the control group, catalase (*CAT*), superoxide dismutase (*SOD*), inducible nitric oxide synthase (*iNOS*), and quinone oxidoreductase 1 (*NQO1*) showed baseline expression, while H_2_O_2_ exposure resulted in pronounced increases in *CAT*, *SOD*, *iNOS*, and *NQO1*, indicating a robust response compared to the Ctl group.

Interestingly, the two concentrations of BB (BB1X and BB2X) significantly increased the expression of *SOD* (*p* < 0.0001 and *p* < 0.01, respectively). Moreover, BB did not significantly increase the expression of the other genes tested. Pre-treatment with BB at the two doses was very effective in downregulating the expression of oxidative genes, except for the BB2X dose for *SOD* (Figure 1B).

### 3.3. BB Extract Alleviates Inflammation Markers

**Figure 2:**
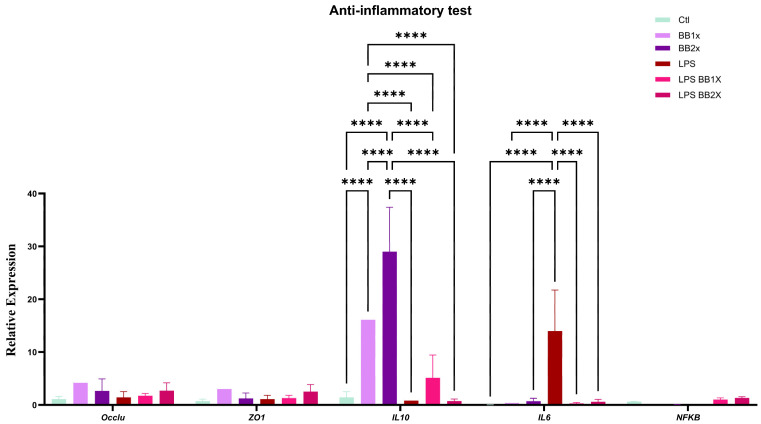
Relative gene expression of *OCCLU*, *ZO1*, *IL10*, *IL6*, and *NFKB* in Caco-2 control cells (Ctl), as well as cells treated with BB at 5 μg/mL (BB1X) and 10 μg/mL (BB2X) concentrations; LPS; and LPS with BB1X (LPS + BB1X) or LPS with BB2X (LPS + BB2X). Data are presented as mean ± SEM, n = 3. Statistical significance is indicated as **** *p* < 0.0001.

In this section, we illustrate the effects of BB (Figure 2) on inflammatory markers and tight junction proteins in an LPS-induced inflammation model using cell culture. The most striking observation is the pronounced dose-dependent upregulation of the anti-inflammatory cytokine *IL10* by BB treatment alone, with BB1X and BB2X inducing approximately 16- and 29-fold increases, respectively, compared to control (*p* < 0.0001). In addition, LPS exposure significantly elevated pro-inflammatory *IL6* expression, approximately 13-fold above control levels (*p* < 0.0001). BB1X and BB2X significantly decreased *IL6*, suggesting that an inflammatory stimulus partially interferes with BB’s ability to upregulate *IL10* and downregulate *IL6*. The expression of genes coding for tight junction proteins (*OCCLU*) and (*ZO1*) was not modified by the LPS or BB treatments.

### 3.4. BB Extract Modulates Colon Gene Expression in Murine Model

**Figure 3:**
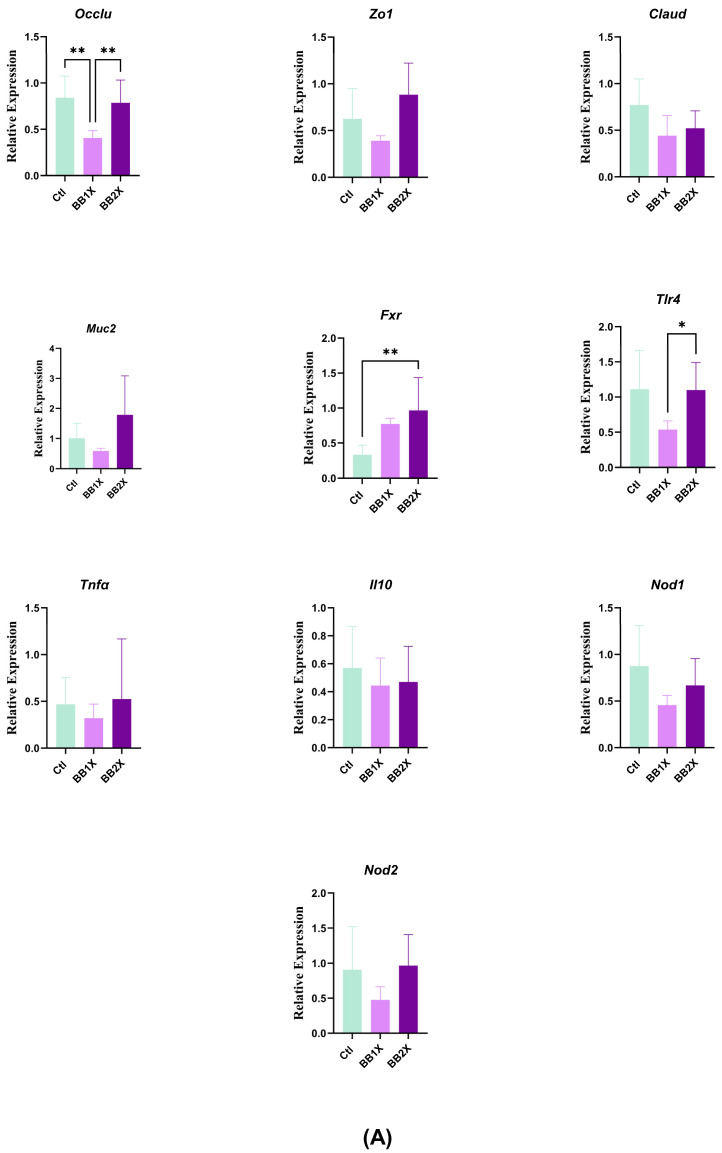

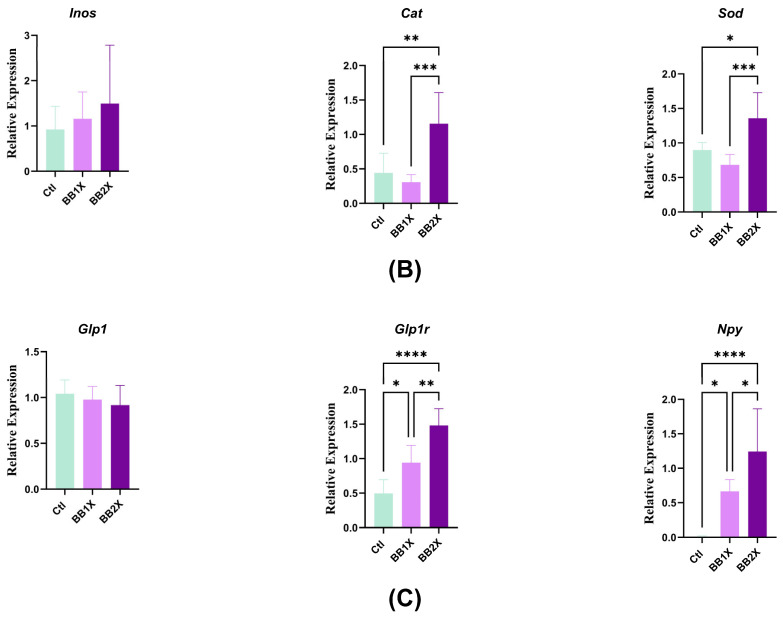
The effects of BB in the colon (n = 8). Bar plots show the relative mRNA expression levels for the following groups: control (Ctl, green), BB1X (2.3 ± 0.26 mg/day dose, light purple), and BB2X (4.8 ± 0.18 mg/day dose, dark purple). Genes are categorised as follows: (**A**) Gut homeostasis genes, (**B**) oxidation genes, and (**C**) gut–brain axis genes. Values represent mean ± SD. Statistical significance was determined via one-way ANOVA (* *p* < 0.05, ** *p* < 0.01, *** *p* < 0.001, and **** *p* < 0.0001).

We studied the effects of the two concentrations of BB (BB1X and BB2X) on the expression of key genes implicated in colon homeostasis in vivo (Figure 3A). The expression of genes coding for the tight junction proteins (*Occlu*, *Zo1*, and *Claud*) was not modified in the BB2X group compared to control mice. However, *Occlu* was significantly downregulated in the BB1X group. Compared to BB2X, BB1X significantly decreased the expression of toll-like receptor 4 (*Tlr4*). *Fxr*—a nuclear receptor involved in bile acid metabolism and intestinal homeostasis—was also increased dramatically in the BB2X group compared to the control, supporting earlier evidence that grape seed polyphenols can modulate nuclear receptor pathways and contribute to gut health [37]. The gene expression levels of the antioxidant enzymes catalase (*Cat*) and superoxide dismutase (*Sod*) were significantly upregulated in the BB2X group (Figure 3B), indicating a dose-dependent enhancement of the antioxidant response, consistent with previous findings that polyphenol-rich extracts such as BB and grape seed extract (GSE) increase antioxidant gene expression and enzyme activities in the colon, thus contributing to protection against oxidative stress and inflammation [21].

Overall, these results indicate that BB—similarly to other oligomeric procyanidin-rich grape seed extracts—exerts dose-dependent antioxidant and barrier-protective effects in the colon.

Notably, we showed that treatment with the two concentrations of BB can upregulate the expression of genes involved in gut–brain signalling, such as neuropeptide Y (*Npy*), in a dose-dependent manner (Figure 3C). Although the glucagon-like peptide gene (*Glp1*) was not upregulated in the colon, its receptor (*Glp1r*) was upregulated significantly in a dose-dependent manner (Figure 3C). These results represent a novel finding that has not been widely reported in the polyphenol literature, suggesting that BB may exert additional neuromodulatory effects in the colon.

### 3.5. Biombalance^TM^ Modulates Ileal Gene Expression in Mice

**Figure 4:**
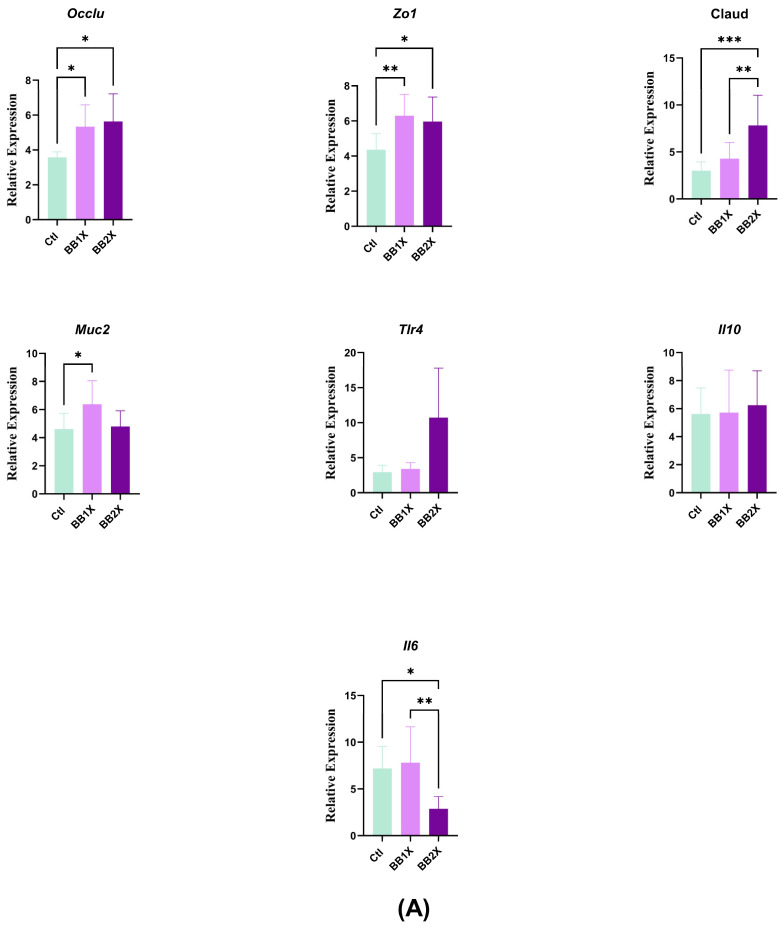

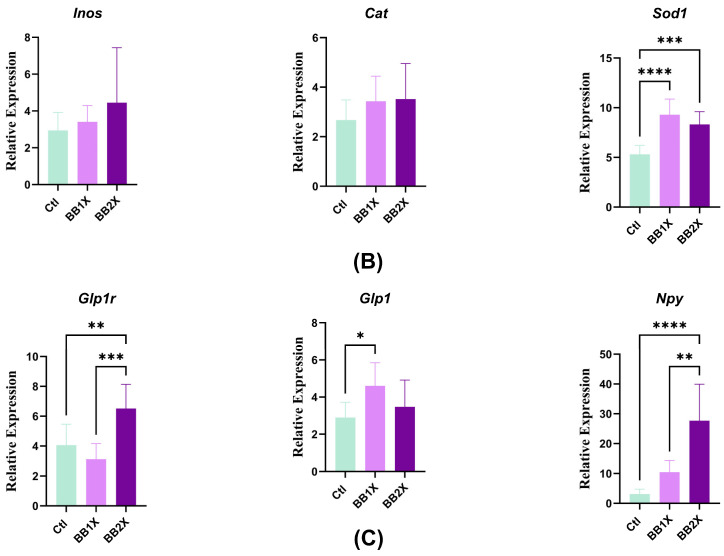
The effects of BB in the ileum (n = 8). Bar plots show the relative mRNA expression levels for the following groups: control (Ctl green), BB1X (2.3 ± 0.26 mg/day dose, light purple), and BB2X (4.8 ± 0.18 mg/day dose, dark purple). Genes are categorised as follows: (**A**) Gut homeostasis genes, (**B**) oxidation genes, and (**C**) gut–brain axis genes. Values represent mean ± SD. Statistical significance was determined via one-way ANOVA (* *p* < 0.05, ** *p* < 0.01, *** *p* < 0.001, and **** *p* < 0.0001).

In contrast to the colon, gene expression of the tight junction proteins *Occlu*, *Zo1*, and *Claud* was significantly upregulated in the ileum. The two BB doses were able to increase *Occlu* and *Zo1* gene expression. In contrast, only the BB2X dose was effective for *Claud* (Figure 4A). In addition, *Muc2* expression was only significantly increased with the BB1X dose, while neither BB dose affected *Il10* and *Tlr4* gene expression (Figure 4A). Concerning oxidative stress-related genes in the ileum, only *Sod1* was significantly upregulated with the two BB concentrations (Figure 4B); this is in contrast to the colon, in which only the BB2X dose was effective for *Sod1* and *Cat* upregulation. The expression of *Npy* and *Glp1r* was upregulated with the BB2X dose (Figure 4)*,* while *Glp1* was upregulated with the BB1X dose (Figure 4C).

### 3.6. BB Extract Modulates Liver Gene Expression in Mice

**Figure 5:**
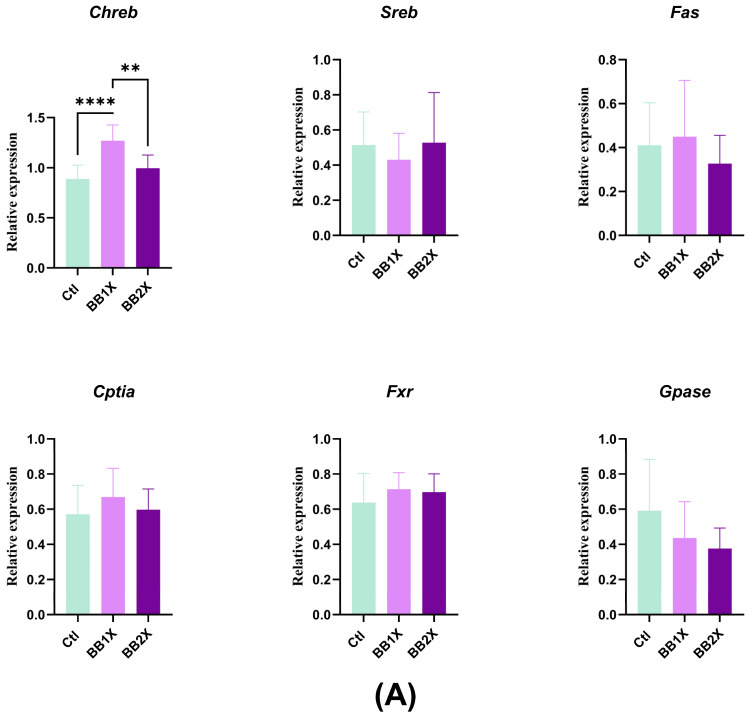

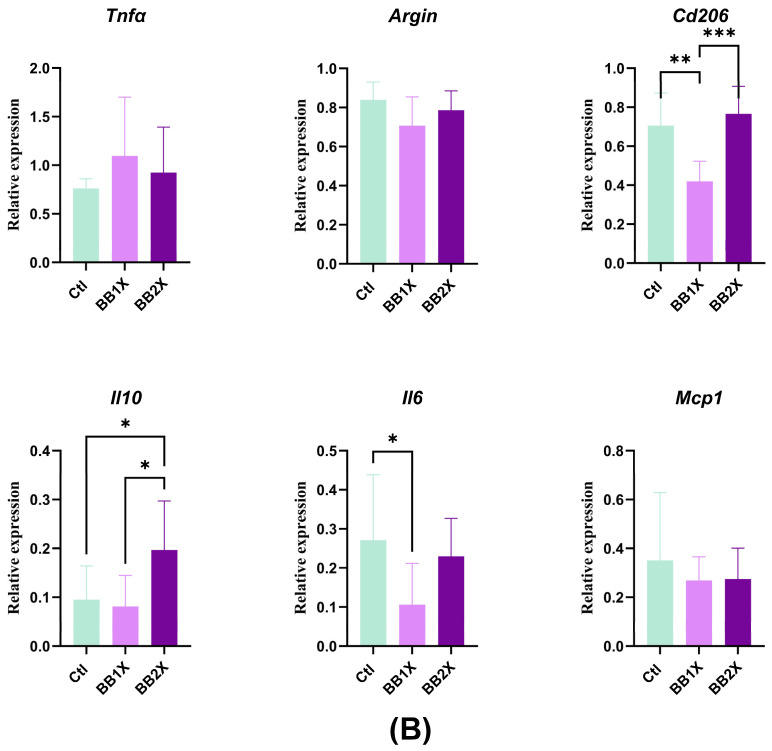

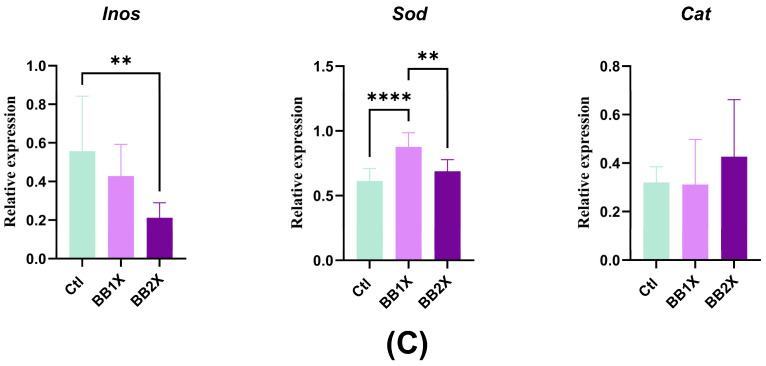
The effects of BB in the liver (n = 8). Bar plots show the relative mRNA expression levels for the following groups: control (Ctl, green), BB1X (2.3 ± 0.26 mg/day dose, light purple), and BB2X (4.8 ± 0.18 mg/day dose, dark purple). Genes are categorised as follows: (**A**) Metabolism genes, (**B**) immunity genes, and (**C**) oxidation genes. Values represent mean ± SD. Statistical significance was determined via one-way ANOVA (* *p* < 0.05, ** *p* < 0.01, *** *p* < 0.001, and **** *p* < 0.0001).

The expression levels of genes implicated in inflammation and oxidative stress, as well as those related to hepatic lipid metabolism, were assessed using real-time PCR to evaluate the effects of BB1X and BB2X. Liver expression of the genes *Il6* and *Cd206* was not altered with the BB2X dose when compared to the control group; however, with the BB1X dose, their expression was significantly lower compared to the control. *Il10* expression was upregulated considerably with the BB2X dose, compared to the control and the BB1X dose. The expression of the genes *Tnfα* and *Mcp1* was not modified (Figure 5A). Moreover, some genes that are implicated in oxidative stress were modulated; for example, *Sod1* expression was significantly upregulated with the BB1X dose, contrary to the BB2X dose, compared to the control (Figure 5B). *Cat* expression was not modified, while that of *iNos* decreased with the BB2X dose (Figure 5B). Taken together, these results indicate the positive anti-inflammatory and anti-oxidative effects of BB extract in the liver. Analysis of hepatic metabolism-related genes revealed only an increased expression in the carbohydrate response element-binding protein (*Chrebp*) with the BB1X dose. In addition, the expression of the sterol regulatory element-binding protein1 (*Srebp1*), carnitine palmitoyltransferase 1 (*Cpt1A*), fatty acid synthase (*Fas*), peroxisome proliferator-activated receptor (*Pparα*), Farnesoid X receptor (*Fxr*), Acetyl-CoA-carboxylase 1 (*Acc1*), and arginase (*Arg1*) did not significantly differ between the groups (Figure 5B). Some of these genes, including *Arg1*, *Fas*, *Srebp1*, and *Il6*, can be considered as markers of liver cytotoxicity [38], indicating that the two BB concentrations used were not cytotoxic.

### 3.7. BB Extract Modulates the Gut Microbiota

The gut microbiota plays a pivotal role in maintaining overall health, including metabolic, immune, and gastrointestinal homeostasis, and has recently been identified as a key regulator of the gut–brain axis. The Illumina MiSeq platform was used to analyse the gut microbiota changes associated with the BB treatments. Alpha diversity—according to the Shannon and Simpson indices—was not significantly modified by the two BB treatments (Supplementary Data S1).

**Figure 6:**
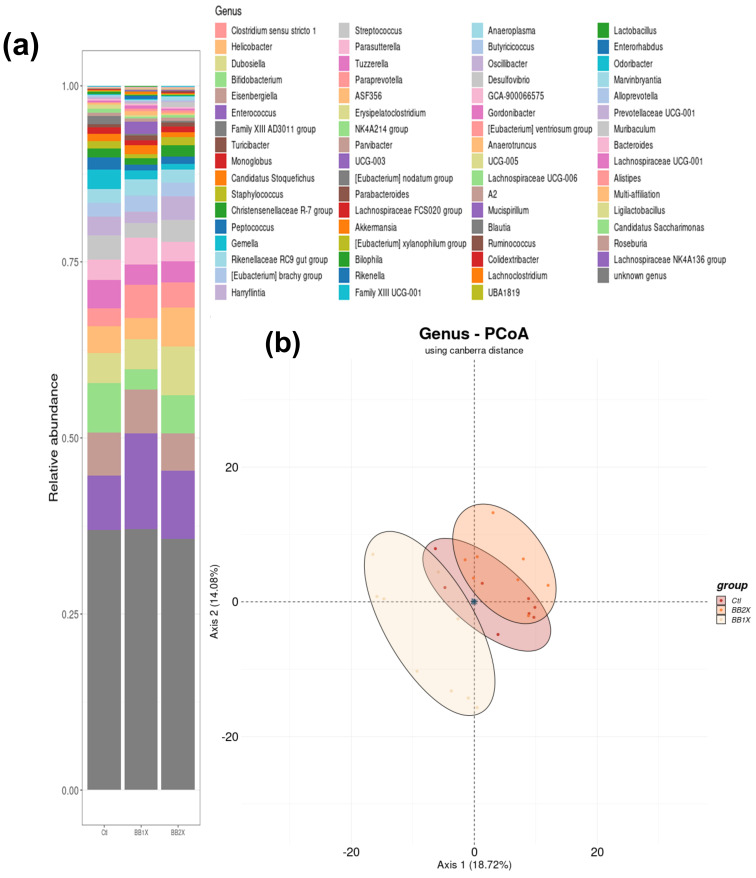
Taxonomic composition of the gut microbiota under Ctl, BB1X, and BB2X treatments at the genus level (n = 8). (**a**) Barplot of the proportions of various taxa in other conditions. (**b**) Principal coordinates analysis (PCoA) using Canberra distance; Axis 1: 18.82%, Axis 2: 15.48%, *p*-value: 0.001.

Subsequently, to evaluate the differences in gut microbiota composition between groups, we performed PCoA at the genus level using the Canberra distance, allowing for illustration of the clustering of gut microbiota among the Ctl, BB1X, and BB2X groups (Figure 6). Ellipses represent the distribution of each group, with key genera indicated near the origin. This plot reveals significant clustering (*p* < 0.01) in the microbial community structure across treatments.

Analysis of the microbiota composition at the phylum level revealed that the treatments did not change the more abundant phyla. However, the phylum Deferribacterota increased significantly only in the BB1X group, while Patescibacteria decreased in the BB1X group. In contrast, Desulfobacterota were only increased in the BB2X group (Supplementary Data S2).

**Figure 7:**
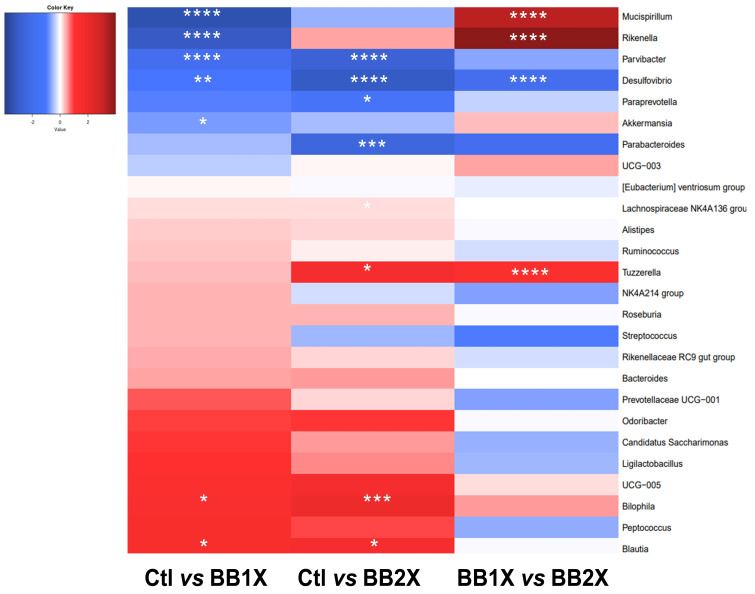
Heatmap illustrating significant dose-dependent alterations in gut bacterial populations following BB administration. Blue indicates an increase in bacterial abundance while red indicates a decrease, with significance levels denoted by asterisks (* *p* < 0.05, ** *p* < 0.01, *** *p* < 0.001, **** *p* < 0.0001).

At the genus level, several bacteria were modulated in the same direction by both BB1X and BB2X doses when compared to the control group (Figure 7). We observed increases in *Parvibacter* and *Desulfovibrio*, and decreases in *Bilophila* and *Blautia*. Moreover, *Mucispirillum*, *Rikenella*, and *Akkermansia* were specifically increased by BB1X, while *Paraprevotella*, *Parabacteroides*, and *Streptococcaceae* were specifically increased by BB2X; contrary to *Lachnospirae* NK4A136 and *Tuzzerella*, which were decreased. Compared to the BB1X dose, *Mucispirillum*, *Rikenella*, and *Tuzzerella* were strongly reduced by BB2X. The differences in microbiota modifications between the two doses may be partially related to the differences in gene expression observed in the colon and ileum.

Interestingly, in the ileum, the altered expression of key genes showed significant positive or negative correlations with the relative abundance of specific bacterial genera, as determined via Spearman’s correlation analysis.

In particular, significant negative correlations were identified between *Blautia* and the expression of *Npy*, *Sod1*, and *Zo1* (r = −0.60, −0.64, and −0.44, respectively). Conversely, the increase in abundance of *Desulfovibrio* following BB treatment was strongly and positively correlated with *Npy*, *Glp1r*, and *Claud* expression (r = 0.80, 0.55, and 0.65, respectively), and negatively correlated with *Il6*, suggesting a beneficial association between *Desulfovibrio* and intestinal function. Complete correlation data are provided in Supplementary Data S3.

### 3.8. PICRUS Results

**Figure 8:**
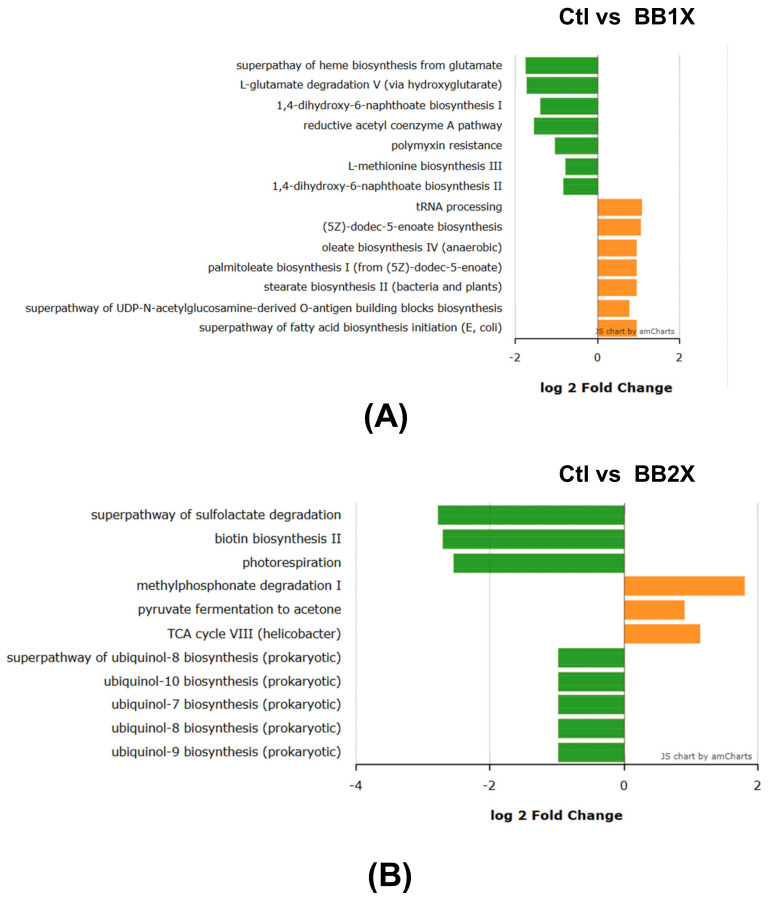

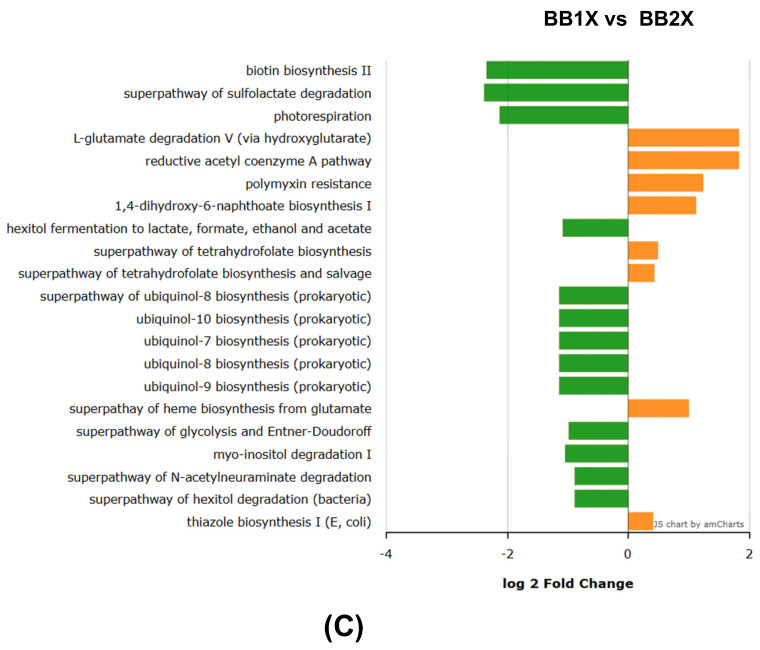
Log2 fold changes in predicted microbiota abundance using KEGG for different experimental treatments (n = 8). (**A**) Ctl vs. BB1X, (**B**) Ctl vs. BB2X, and (**C**) BB1X vs. BB2X. Green—significantly increased; orange—significantly decreased (*p* < 0.05).

Comparative KEGG pathway analysis demonstrated distinct dose-dependent alterations across the experimental groups. Relative to control (Ctl), the BB1X treatment (Figure 8A) principally enhanced pathways associated with glutamate degradation (L-glutamate degradation V (via hydroxyglutarate) and heme biosynthesis from glutamate), energy metabolism (reductive acetyl coenzyme A pathway), and L-methionine biosynthesis, while markedly suppressing fatty acid biosynthesis cascades (palmitoleate/stearate/oleate biosynthesis) and tRNA processing. The higher-dose BB2X treatment (Figure 8B) induced more pronounced modifications, significantly upregulating the pathways associated with sulfolactate degradation (conversion of glutamate into oxoglutarate), biotin and ubiquinol biosynthesis (7 to 10), while downregulating those relating to methylphosphonate degradation and fermentation of pyruvate to acetone. A direct comparison between BB1X and BB2X (Figure 8C) revealed that the higher dose further amplified pathways related to biotin biosynthesis, sulfolactate degradation, and ubiquinol biosynthesis, while simultaneously reducing the L-glutamate degradation, heme biosynthesis from glutamate, polymyxin resistance, and tetrahydrofolate biosynthesis pathways. These shifts in metabolic potential suggest that BB exerts selective pressure on microbial communities, promoting beneficial pathways while suppressing potentially deleterious metabolic processes, consistent with our recent findings that polyphenol-rich extracts can systematically restructure gut microbial functionality [21].

## 4. Discussion

The present study presented BB as a multifunctional keystone food supplement.The in vitro analyses as well as the in vivo model proved the antibacterial, antioxidant and anti-inflammatory effects of BB.

In a previous study, we demonstrated the remarkable antioxidant effect of BB in vitro (DPPH radical scavenging IC50 = 24.66 ± 0.02 M TE) [21], attributed to its high content of catechins, epicatechins, and B-type procyanidin polymers, with the degree of polymerisation influencing both antioxidant capacity and biological effects [21] BB acts as a potential natural antimicrobial, with particular efficacy against certain Gram-positive bacteria such as *S. aureus*, and is also able to inhibit Gram-negative bacteria such as the pathogen *H. pylori* [11] with an MIC similar to that of antibiotics (1 to 2 mg/mL). It has been reported that muscadine GSEs were effective in inhibiting *H. pylori* in vitro, and it was suggested that the polyphenol contents in such extracts (hydrolysed, condensed tannins and flavan-3-ols) [38]—similar to those found in BB—may contribute to the antimicrobial activities reported in the literature [38]. Although still a subject of debate, some data from the literature suggest an association between *H. pylori* and SIBO; for example, by decreasing gastric pH, *H. pylori* can affect the proximal small intestine (duodenum) and may predispose individuals to SIBO [39,40]. Our study demonstrated that BB could inhibit some of the bacteria involved in SIBO, including *Enterococcus*, *Streptococcus*, and *Staphylococcus* [12]. This suggests that BB could simultaneously target multiple gastrointestinal pathogens and related disorders, opening the door to the concept that this GSE, rich in OPCs, could serve as a coadjuvant in antibiotic therapy for *H. pylori* and SIBO [41]. BB also moderately inhibits *P. gingivalis* and *S. mutans*, oral pathogens implicated in periodontitis and caries, respectively. *S. mutans* has also been linked to extra-buccal disorders, with a recent study demonstrating that the imidazole propionate produced by *S. mutans* can drive Parkinson’s disease [42]. In addition to the antibacterial effects of BB on oral pathogens, its proanthocyanidin content has also been described as strengthening collagen-based tissues, increasing collagen synthesis, and enhancing the remineralisation process of human teeth [43]. Hence, depending on the final formulation chosen for the use of BB as a food supplement, this GSE could have an inhibitory impact on pathogens from the mouth to the small intestine, and may be combined with antibiotics to exhibit a complementary or synergistic effect, potentially establishing promising additional strategies to cope with bacterial issues [44].

The protective potential of BB was further assessed in Caco-2 cells subjected to H_2_O_2_-induced oxidative stress or LPS-induced inflammation. The BB extract protected cells against oxidative damage without cytotoxicity, and significantly increased *IL10* expression while decreasing *IL6* expression after LPS challenge. These results demonstrate its apparent cytoprotective effects against oxidative stress and inflammation—a finding similar to that of Laskowska et al. [45]. These results highlight the potent bioactivity of BB, especially given that comparable levels of protection in prior studies using other phenolic extracts typically required higher concentrations (e.g., in the milligram range) [46,47]. The original composition of BB, which combines epicatechin and B-type procyanidin dimers, procyanidin trimers, and tetramers, may have direct antioxidant effects, as confirmed by the in vitro results [5]. It is worth noting that the gut microbiota extensively ferments polyphenols that reach the colon, which may result in differences in an in vivo model. We confirmed the results of a previous study, in which BB demonstrated anti-inflammatory and antioxidant effects in both the ileum and colon in a DSS-induced murine colitis model [21]. Most complex, high-molecular-weight polyphenols pass through the digestive tract without being absorbed, and are only broken down by gut bacteria into smaller, bioactive compounds once they reach the colon [48]. The compositional diversity of BB extract [21] may offer good potential for delivering polyphenols which are active and absorbed in different parts of the gastrointestinal tract.

In the present study, we supplemented the diet of healthy mice with two different doses of BB and observed their effects on gene expression in various parts of the GIT. In the ileum, the BB1X and BB2X doses induced upregulation of genes encoding tight junction proteins (Occlu and Zo1), which are pivotal in maintaining the integrity of the intestinal epithelial barrier, compared with the colon [49].

The same results were observed in our previous study, in which BB was administered by gavage to a healthy group of Balb/c mice: Occlu and Zo1 gene expression were significantly upregulated in the ileum (but not the colon) of the healthy control group treated with BB compared with non-treated mice [21]. These results suggest that a specific class of polyphenols present in the BB can modulate the expression of tight junction proteins in the upper part of the GIT in a basal state. The direct effect may be attributable to less complex polyphenols such as monomers, as a study performed by Yang et al. (2021) indicated a correlation between a lower degree of polymerisation of procyanidins and higher bioactivity in the upper GIT [50].

Proanthocyanidins have been shown to exhibit anti-inflammatory and antioxidant properties, playing beneficial roles in mitigating oxidative stress and inflammation in the liver [5,51]. Interestingly, regarding liver gene expression evaluated in this study, both doses had a specific effect: the 1X dose altered the expression of specific genes that were not altered by the 2X dose (*Chrebp*, *Cd206*, *Il6*, and *Sod*). In some cases, the opposite was also true: the 2X dose modified the expression of genes that were not modified by BB1X (*Il10* and *iNos*). In our previous study, in a basal state, the Balb/c mice force-fed with BB did not show any modifications in the expression of *Chrebp*, *iNos*, and *Il6* genes. In this context, it seems that BB in the diet naturally alters the expression of different genes in the liver, which are not modified when administered by gavage. Moreover, in the present study, with a healthy mouse model, neither doses had an impact on the expression of genes in the liver that are involved in metabolism. On the contrary, when inflammation is triggered—as in the DSS model previously utilised by our group—BB was able to counteract the decrease in *Chrebp* induced by inflammation [21].

A novel finding of our study is the stimulatory effect of BB on Npy gene expression; in particular, Npy was upregulated in the ileum and colon in a dose-dependent manner. NPY is a 36-amino acid peptide that serves as a neurotransmitter and neuromodulator; it is expressed in both the central and peripheral nervous systems, where it regulates diverse physiological and behavioural processes such as energy homeostasis, stress response, and emotional regulation [52]. In the gastrointestinal system, NPY is secreted by enteric neurons and enteroendocrine cells, playing crucial roles in gastrointestinal motility, secretion, and local immune responses, and being integral to gut–brain axis communication and systemic metabolic control [52,53].

Research has shown strong associations between the structure of the gut microbial community and NPY expression, as well as cognitive impairments mediated by the microbiota–gut–brain axis [54,55]. A pivotal investigation demonstrated that transferring gut microbes from individuals with ulcerative colitis led to reduced *NPY* expression in the colon and, at the same time, gave rise to anxiety- and depression-like behaviours in mice [55]. This provides one of the clearest pieces of evidence linking intestinal NPY activity to behavioural and cognitive changes.

Moreover, the administration of BB2X increased the expression of *Glp1* in the ileum.

Endocrine L-cells secrete this peptide in the gut, which are located primarily in the distal small intestine and colon, in response to nutrient ingestion [56]. GLP-1 is a pivotal incretin hormone that regulates glycaemic homeostasis, appetite, gut motility, and barrier integrity, which exerts its multi-beneficial effects through the specific GLP-1 receptor (GLP-1r) [56,57]. Polyphenols can both directly act on L-cells and indirectly enhance GLP-1 secretion by modulating gut microbial composition and metabolic outputs, including the production of short-chain fatty acids and bile acid signalling via receptors such as TGR5 and FXR [58].

In accordance with our study, Kartinah et al. [59] demonstrated the potential of *Hibiscus sabdariffa* (rich in anthocyanins) to induce *Glp1* expression in the ileum of rats. In humans, ingestion of 3 g of cinnamon (but not 1 g) increased the plasmatic GLP-1 concentration [60].

Physiologically, most of the actions of GLP-1 are exerted locally in the gut via GLP-1r, including those expressed by L-cells, gut lymphocytes, and, in particular, numerous intestinal nerve fibres. Consequently, activation of GLP-1r may be associated with both local effects and central transmission of signals via sensory afferents of the vagus nerve [61]. Interestingly, the two doses of BB increased GLP-1r expression in the colon, while only BB1X increased it in the ileum. Moreover, deletion of *Glp1r* (*Glp1r*^−/−^ mice) led to microbiota dysbiosis, as well as increased sensitivity to inflammation-related injury [62].

Therefore, the microbiota modifications produced by the BB treatments can be correlated with changes in colon gene expression. In fact, significant negative correlations were observed between Blautia abundance and *Npy*/*Sod1*/*Zo1* expression (r = −0.6, −0.64, and −0.44, respectively), indicating an adverse effect of *Blautia* on gut homeostasis. A recent article revealed correlations between the abundance of *Blautia* and neurological diseases, as well as Down syndrome and Alzheimer’s Disease [63,64]. Moreover, this study demonstrated the anti-*Blautia* effect of BB in both in vivo and in vitro models. BB is the first described OPC with an anti-*Blautia* effect. In addition, the increase in *Desulfovibrio* observed after BB treatment correlated positively with *Npy*/*Glp1r*/*Claud* expression (r = 0.8, 0.55, and 0.65, respectively) and negatively with Il6 expression, indicating a positive effect of *Desulfovibrio* [65].

The observed effects of BB on the expression of critical incretins, such as GLP-1 and NPY, are promising and suggest that modulation of the gut–brain axis by a natural, beneficial prebiotic could offer substantial opportunities for future applications.

In our study, the light impact of BB on bacterial diversity did not reflect the strong metabolic functional potential on the microbiome. When focusing on metabolic alterations, it should be underscored that interspecies interactions and cooperative dynamics within the gut microbiota are more pivotal to host health than the mere compositional profile of microbial communities, as these metabolic networks directly modulate immune responses, metabolic homeostasis, and intestinal barrier function [66].

As mentioned above and reported in the literature, delivery by gavage or via diet produced distinct effects in murine models [67], with differences in microbiota analysis between the two administration routes. Notably, the gavage protocol led to more substantial alterations in microbial composition, despite equivalent total exposure to the compound. Unlike the prior experiment, an increase in beneficial taxa—such as Roseburia—was not observed in the present study [21]. Nevertheless, the gavage approach, which more closely mimics oral supplementation with a capsule and minimises dietary interactions, appears to be more effective in modulating the gut microbiota than dietary administration [67]. The drawback of the gavage method is the acute stress it induces in animals, which may further influence physiological parameters and confound the interpretation of microbiota-related outcomes [68].

Although BB administration modestly alters the microbiota, the PICRUS analysis indicated that BB treatment modified several relevant metabolic pathways. As in our previous study [21] using the same dose of BB2X, we observed a significant increase in the biosynthesis of ubiquinol (coenzyme Q7 to Q10). This suggests a greater prevalence of bacteria capable of adapting to more varied microaerobic conditions, such as those in proximity to the host cells [69]. Ubiquinols are membrane-fatty-soluble metabolites with strong antioxidant capacities, and we have previously postulated that bacterial extracellular vesicles have the potential to deliver CoQ to intestinal cells [21]. Other interesting pathways modified following BB administration are related to the metabolic transformation/degradation of glutamate. In fact, as in our previous study [21], the sulfolactate degradation pathway increased under the BB2X treatment, which has been implicated in the conversion of L-glutamate into oxoglutarate. This pathway is present in the sulphate-reducing bacteria (SRB) members, such as *Desulfovibrio*, which are the most frequent SRB present in the colon [70]. This suggests that *Desulfovibrio*, which also increased with BB2X treatment, may be involved in glutamate degradation. Two other pathways—heme biosynthesis from glutamate and L-glutamate degradation V (via hydroxyglutarate)—are also involved in the degradation of glutamate and were increased under the BB1X treatment. The possible conversion and/or degradation of glutamate by gut microbiota members may be of functional interest.

The primary sources for luminal glutamate are dietary protein and flavour-enhancing food additives, such as monosodium glutamate (E621) [71]. Various clinical studies have reported correlations between elevated circulating glutamate concentration, obesity, and T2 diabetes in cohorts from different countries [72,73]. Recently, Han et al. (2025) [74] proposed the mechanism that people living with obesity have a lower abundance of bacterial species catabolising glutamate, leading to glutamate accumulation in the intestine. In relation to these findings, BB would be of great interest in the context of obesity due to its capacity to increase the abundance of bacteria that are implicated in glutamate degradation. Moreover, the functional superpathway related to heme biosynthesis from L-glutamate (via hydroxyglutarate), as identified in our study, has been associated with a decreased risk of asthma. Through this pathway, bacteria can transform amino acids into SCFAs, among other products [75]. Accumulating evidence indicates that alterations in the gut microbiota can significantly influence brain glutamate levels [76]; in fact, glutamate—a ubiquitous neurotransmitter—can become a potent excitotoxin when present in excessive levels, predisposing individuals to neuroinflammation and neurotoxicity [77]. Preclinical studies have shown that diets high in sodium glutamate can induce depressive behaviours in rodents, and that microbial populations can either facilitate or limit glutamate uptake [77]. In this context, the administration of BB can help to shape microbial populations that facilitate glutamate degradation, including *Desulfovibrio* and possibly other genera. Furthermore, considering the capacity of BB to increase the intestinal expression of tight junction proteins (Claudin, Occludin, ZO1), it could help to inhibit glutamate absorption. We can postulate that, due to these properties, BB may be of interest in the context of depression and other brain disorders related to excessive glutamate. However, complementary studies are necessary for further validation.

Despite the absence of behavioural assessments to evaluate the psychological impacts of gene modulation and the lack of quantification of incretins and metabolites in either brain or plasma, this study introduces several innovative features that may advance our understanding of the mechanisms by which OPCs influence health. The findings of this study open up new perspectives on the effects of grape seed extracts, particularly regarding the gut–brain axis and metabolism. To strengthen these observations, it will be necessary to confirm the effects of this extract in various mouse models: first, in an infectious model such as one involving *H. pylori*; second, in an obesity model to further assess the GLP-1 modulation observed in this study; and third, in a model of mood disorders to evaluate its potential influence on gut–brain axis communication.

## 5. Conclusions

In conclusion, BB—a GSE rich in oligomeric procyanidins—exhibits multifunctional bioactivity, including selective in vitro antibacterial properties against key bacterial pathogen strains, such as *S. aureus* and *H. pylori*. Moreover, in the Caco-2 cell line, BB produced antioxidant and anti-inflammatory effects in response to H_2_O_2_ or LPS, respectively. Finally, in an in vivo model, two different BB doses were found to modulate the gut microbiota and have positive impacts on gut homeostasis, oxidation, and specific inflammatory markers. Due to its potent antioxidant and anti-inflammatory effects in vitro and in vivo, as well as its antibacterial properties, BB has significant health potential for buccal care and may serve as an adjuvant against *H. pylori* or as an anti-SIBO ingredient. BB also positively impacts gut homeostasis and signalling pathways by increasing the expression of genes such as *Occlu*, *Glp1*, and *Npy.* Finally, BB can shape gut microbial populations (e.g., *Desulfovibrio*), which can facilitate glutamate degradation, thereby potentially affecting obesity, type 2 diabetes, and even depression.

## Figures and Tables

**Table 1:** Minimal inhibitory concentrations (MIC) of BB against the indicated bacterial strains. Values are presented as the lowest concentration (in mg/mL) that completely inhibited visible bacterial growth after 24 or 48 h of incubation. Each result represents the mean of three independent experiments—abbreviations: NC, Not Conclusive, which means the MIC is greater than 32 mg/mL.

Target Bacteria	MIC (mg/mL)
*Staphylococcus aureus* CIP 20256	1
*Kocuria rhizophila* ATCC 10240	1
*Helicobacter pylori* p12	1
*Helicobacter pylori* 7.13	2
*Blautia coccoides* DSM 935	8
*Listeria monocytogenes* CIP 82110	16
*Listeria innocua* CIP 106065	16
*Enterococcus faecalis* CIP 76117	16
*Streptococcus mutans* ATCC 35668	16
*Porphyromonas gingivalis* ATCC 33277	16
*Pseudomonas aeruginosa* CIP 76110	16
*Erwinia carotovora* CECT 225	16
*Fusobacterium nucleatum* ATCC 25586	NC
*Escherichia coli* CIP 7624	NC
*Cutibacterium acnes* DSM 1897	NC
*Pediococcus acidilactici* DSM 20284	NC
*Lactobacillus plantarum* 299v	NC
*Akkermansia muciniphila* DSM 22959	NC

## Data Availability

The original contributions presented in this study are included in the article and Supplementary Materials. Further inquiries can be directed to the corresponding authors.

## Supplementary Materials

The following supporting information can be downloaded at: https://www.mdpi.com/article/10.3390/antiox14121484/s1, Table S1: BB composition. Figure S1: Effects of BB on microbiota diversity. Figure S2: Log2 fold changes in predicted microbiota abundance for different experimental treatments. Figure S3: Spearman’s rank correlation analysis between the relative abundances of *Blautia*, *Desulfovibrio*, and *Mucispirillum* and the expression levels of ileal genes.

## Abbreviations

ARG-1	arginase 1
CAT	catalase
ChREBP	carbohydrate-responsive element-binding protein
CLAUD	claudin
DPPH	2,2-Diphenyl-1-picrylhydrazyl
FXR	Farnesoid X Receptor
GLP-1	glucagon-Like Peptide-1
GLP-1r	glucagon-Like Peptide-1 receptor
HPRT	hypoxanthine phosphoribosyl transferase
IL-6	interleukin 6
IL-10	interleukin 10
iNOS	inducible nitric oxide synthase
MUC2	mucin 2
NOD-1	nucleotide-binding oligomerisation domain-containing protein 1
NOD-2	nucleotide-binding oligomerisation domain-containing protein 2
NPY	neuropeptide Y
OCCLU	occludin
SOD	superoxide dismutase
TLR4	toll-like receptor 4
TNF-α	tumour necrosis factor α
ZO-1	zonula occluens-1
