# Supplementary figures and images for: BiombalanceTM: A Specific Oligomeric Procyanidin-Rich Grape Seed Extract as Multifunctional Ingredient Integrating Antibacterial, Antioxidant, and Anti-Inflammatory Activities with Beneficial Gut–Brain Axis Modulation
Source: Antioxidants (Basel). 2025 Dec 10;14(12):1484. doi: 10.3390/antiox14121484 (PMC12729549; doi:10.3390/antiox14121484)

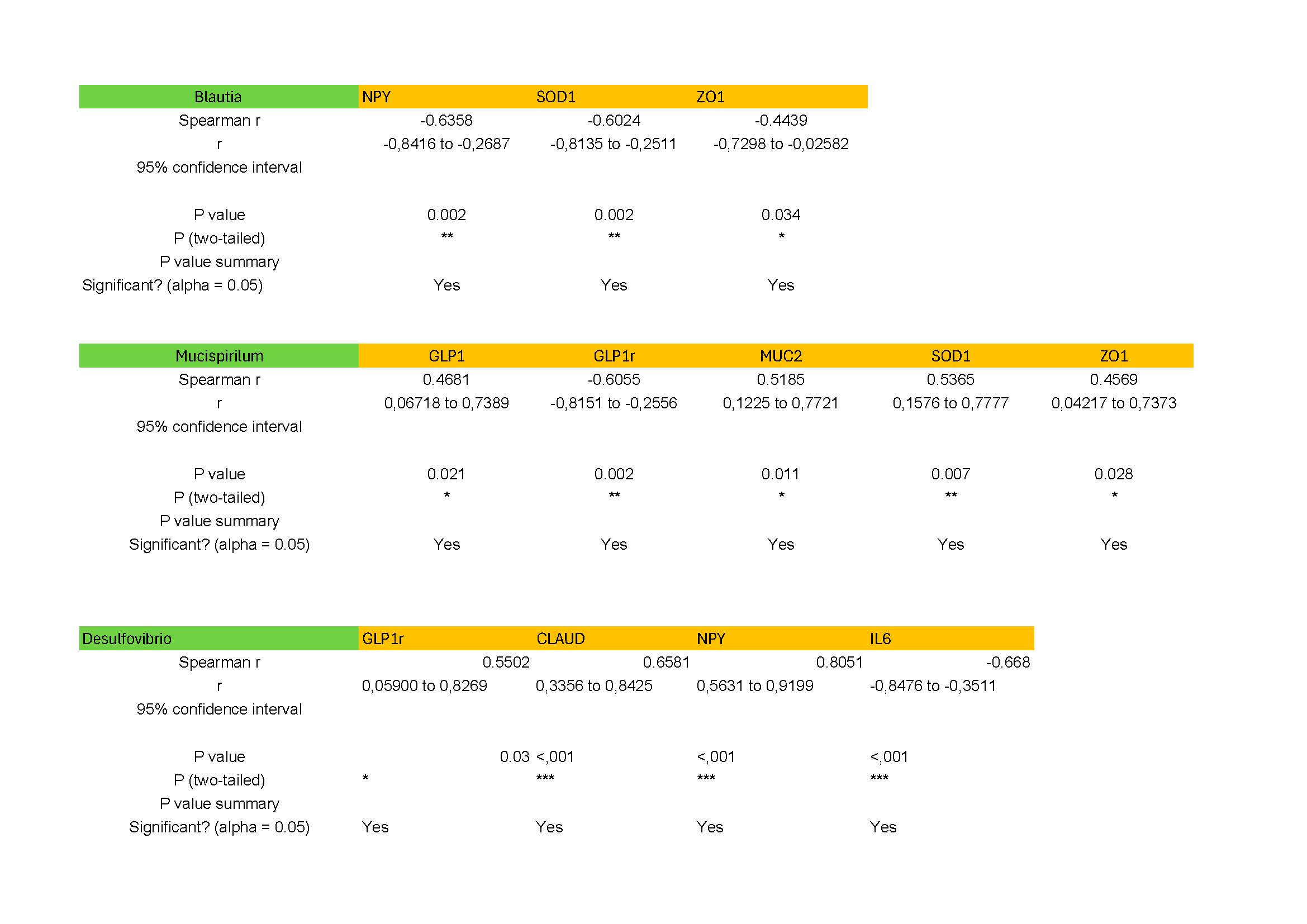

Supplement: Supplementary file 1 [file antioxidants-14-01484-s001.zip › Figure S3.jpg]
